# Biochemical Targets and Molecular Mechanism of Matrine against Aging

**DOI:** 10.3390/ijms241210098

**Published:** 2023-06-14

**Authors:** Kaiyue Sun, Yingzi Zhang, Yingliang Li, Pengyu Yang, Yingting Sun

**Affiliations:** College of Veterinary Medicine, Shanxi Agricultural University, Taigu, Jinzhong 030801, Chinasxxncc@163.com (Y.L.);

**Keywords:** matrine, aging, network pharmacology, PARP1/NAD^+^

## Abstract

The aim of this study is to explore the potential targets and molecular mechanism of matrine (MAT) against aging. Bioinformatic-based network pharmacology was used to investigate the aging-related targets and MAT-treated targets. A total of 193 potential genes of MAT against aging were obtained and then the top 10 key genes (cyclin D1, cyclin-dependent kinase 1, Cyclin A2, androgen receptor, Poly [ADP-ribose] polymerase-1 (PARP1), histone-lysine N-methyltransferase, albumin, mammalian target of rapamycin, histone deacetylase 2, and matrix metalloproteinase 9) were filtered by the molecular complex detection, maximal clique centrality (MMC) algorithm, and degree. The Metascape tool was used for analyzing biological processes and pathways of the top 10 key genes. The main biological processes were response to an inorganic substance and cellular response to chemical stress (including cellular response to oxidative stress). The major pathways were involved in cellular senescence and the cell cycle. After an analysis of major biological processes and pathways, it appears that PARP1/nicotinamide adenine dinucleotide (NAD^+^)-mediated cellular senescence may play an important role in MAT against aging. Molecular docking, molecular dynamics simulation, and in vivo study were used for further investigation. MAT could interact with the cavity of the PARP1 protein with the binding energy at −8.5 kcal/mol. Results from molecular dynamics simulations showed that the PARP1-MAT complex was more stable than PARP1 alone and that the binding-free energy of the PARP1-MAT complex was −15.962 kcal/mol. The in vivo study showed that MAT could significantly increase the NAD^+^ level of the liver of d-gal-induced aging mice. Therefore, MAT could interfere with aging through the PARP1/NAD^+^-mediated cellular senescence signaling pathway.

## 1. Introduction

The number of over 60-year-olds increased rapidly globally, and it is estimated that the population of over 60-year-olds will reach 2 billion in 2050; meanwhile, the number of over 80-year-olds increased significantly. Moreover, notably, the rate of the aging population increased fastest in low- and-middle-income countries around the world [1]. With aging, the risk factors of aging-related diseases increases, such as osteoporosis, Alzheimer’s disease (AD), obesity, diabetes, and cardiovascular disorders [2]. Even though the global average life expectancy was 72, the healthy lifespan was only 63 years in 2016 [1]. Therefore, aging is a public problem and aging-related diseases have attracted worldwide attention. Previous studies show that the important hallmarks of aging include deregulated nutrient sensing, cellular senescence, mitochondrial dysfunction, genomic instability, stem-cell exhaustion, and so on. The current mainstream interventions to delay aging-related phenotypes and diseases include oral administration of drugs, exercise, a healthy diet, and so on [2]. Our goal is to search for candidate compounds with anti-aging effects and further understand how they affect aging. 

*Sophora flavescens* is a famous medicine in traditional Chinese medicine (TCM), with its root having been used to treat fever, hematochezia, vulvar swelling, and inflammatory diseases [3]. Martine (MAT) is the major alkaloid extracted from *Sophora flavescens*. It has been reported that MAT has numerous pharmacological activities, such as anti-depression, colitis-protective activity, antibacterial and antiviral activity, hepatorenal protective activity, antidiabetic, antitumor, and so on [4,5,6,7,8,9,10]. In addition, MAT plays an important role in delaying aging-related diseases. MAT not only could produce a neuroprotective effect by inhibiting acetylcholinesterase and butyrylcholinesterase activities to prevent AD [11] but could also improve cardiac fibrosis by affecting the ATF6-signaling pathway in diabetic cardiomyopathy [12]. In the previous study, we found that MAT could protect against aging induced by d-gal by inhibiting cellular senescence [13]. However, current knowledge about the underlying mechanism of MAT against aging has not been addressed yet, and its system-level mechanism warrants further investigation. 

Network pharmacology analysis is one of the most common methods which uses computers, and high-throughput omics data analysis to study the pharmacology of TCM. We can analyze the links among compounds from TCM, potential targets, and diseases, and obtain information on the system-level mechanism of active compounds [14]. Molecular docking is an important tool for understanding how the active compounds interact with the predicted targets so that it can be used to verify the accuracy of these targets [15]. However, due to the limitation of molecular docking, molecular dynamic (MD) stimulation is used to further validate and refine the modeled complex. The thermodynamics and kinetics related to drug-target recognition and binding could be more accurately estimated by MD stimulation [16,17]. Therefore, we employed the network pharmacology analysis method and the molecular docking technology combined with MD stimulation to explain the action of the mechanism of MAT against aging in this study.

## 2. Results

### 2.1. Analysis of MAT and Collection of Its Potential Targets

**Figure 1:**
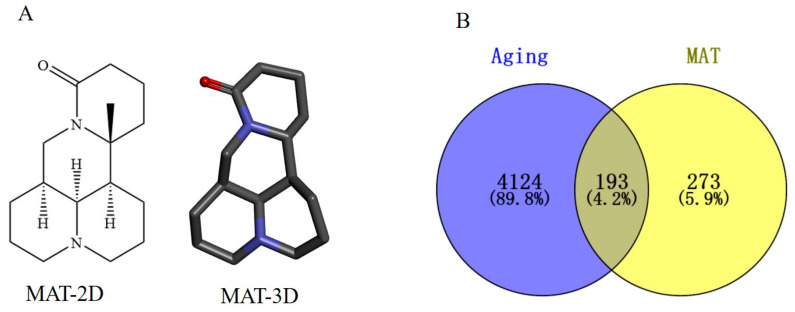
The structure of MAT and acquisition of common targets of MAT against aging. (**A**) two-dimensional (2D) and three-dimensional (3D) structure of MAT. (**B**) Potential targets of MAT against aging.

The two-dimensional and three-dimensional structures were shown in Figure 1A, and the pharmacological and molecular properties of MAT were collected from the TCMSP database (Table 1). The molecular weight of MAT is 248.41. The oral bioavailability (OB) (63.77%), half-life (HL) (6.69), the Ghose-Crippen octanol-water partition coefficient (AlogP) (1.42), topological polar surface area (TPSA) (23.55), and drug-likeness (DL) (0.25) of MAT proved its high bioavailability and suitable DL property, and the blood-brain barrier (BBB) (1.52) of MAT showed its strong penetrating. A total of 466 potential targets were obtained by combining and removing duplicate targets from the Pharmmapper database, the SwissTargetPrediction database, and the SuperPred Database. 

### 2.2. Collection of Cellular Aging-Related Genes

A total of 3672 targets were discovered using GeneCards (GIFtS > 40), 227 targets were identified using OMIM, and 1261 targets were obtained from the NCBI GENE database. The three different databases were combined and duplicates were removed. Analysis of data in the three different databases identified 4317 targets associated with cellular aging. Next, the MAT targets were intersected with the aging genes to obtain the overlapped genes and a Venn diagram was plotted (Figure 1B).

### 2.3. Protein–Protein Interaction Network Analysis

**Figure 2:**
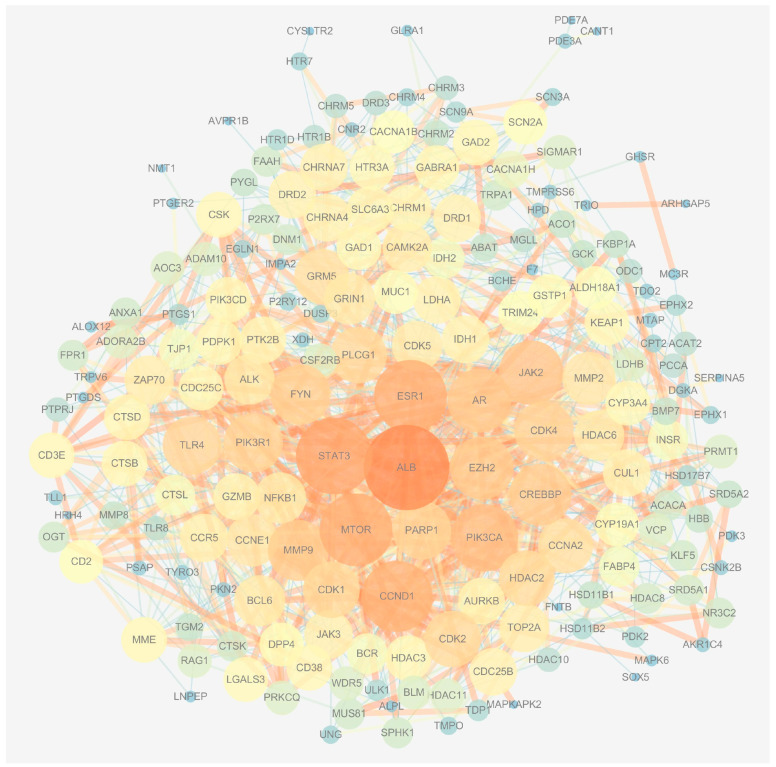
Construction of the PPI network.

**Figure 3:**
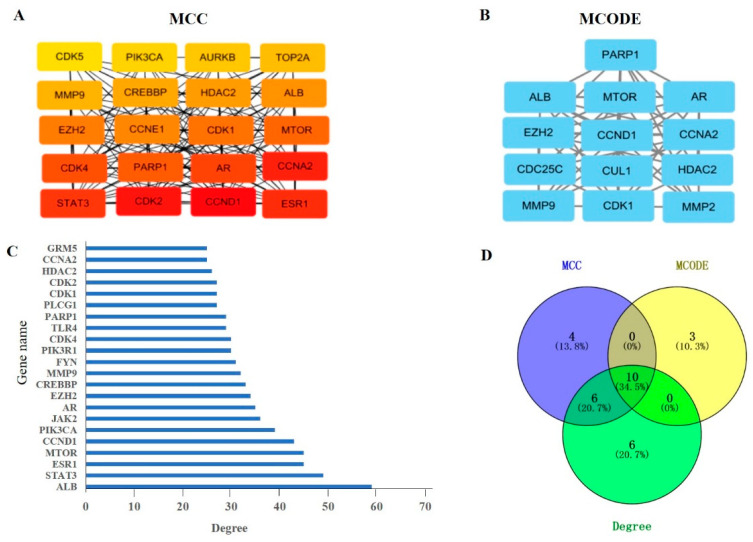
The determination of the key genes of MAT against aging. (**A**) The top 20 genes were obtained according to the score calculated by the MCC method using Cytoscape software. (**B**) A total of 13 candidate genes were identified by the MCODE method using Cytoscape software. (**C**) The genes with a DC value greater than twice the median of the DC were selected according to the score calculated by the degree method. (**D**) The Venn diagram displays the top 10 key genes; these genes were obtained by the intersection of the three methods (MCC, MCODE, and degree) and they are CCND1, CDK1, CCNA2, AR, PARP1, EZH2, ALB, mTOR, HDAC2, and MMP9.

A total of 193 potential genes were obtained after the intersection and then uploaded to the String database to conduct the Protein–Protein Interaction (PPI) network. The PPI network was imported into Cytoscape software (version 3.7.1), and the topological parameters of the network were analyzed. The results showed that the network had 191 nodes and 1115 edges total (Figure 2). The top 20 genes were filtered by the maximal clique centrality (MMC) algorithm, and the network had 20 nodes and 150 edges, as shown in Figure 3A. The first gene cluster with the highest scores, containing 9 scores, 13 nodes, and 54 edges was obtained by using the molecular complex detection (MCODE) algorithm (Figure 3B). There were 22 genes whose gene degree (DC) was greater than twice the median of the DC (Figure 3C). The top 10 key genes were filtered after the intersection of the genes obtained from three methods; these are cyclin D1 (CCND1), cyclin-dependent kinase 1 (CDK1), Cyclin A2 (CCNA2), androgen receptor (AR), Poly [ADP-ribose] polymerase-1 (PARP1), histone-lysine N-methyltransferase (EZH2), albumin (ALB), mammalian target of rapamycin (mTOR), histone deacetylase 2 (HDAC2), and matrix metalloproteinase 9 (MMP9) (Figure 3D). 

### 2.4. Biological Processes and Pathway Enrichment Analysis of the Key Genes

**Figure 4:**
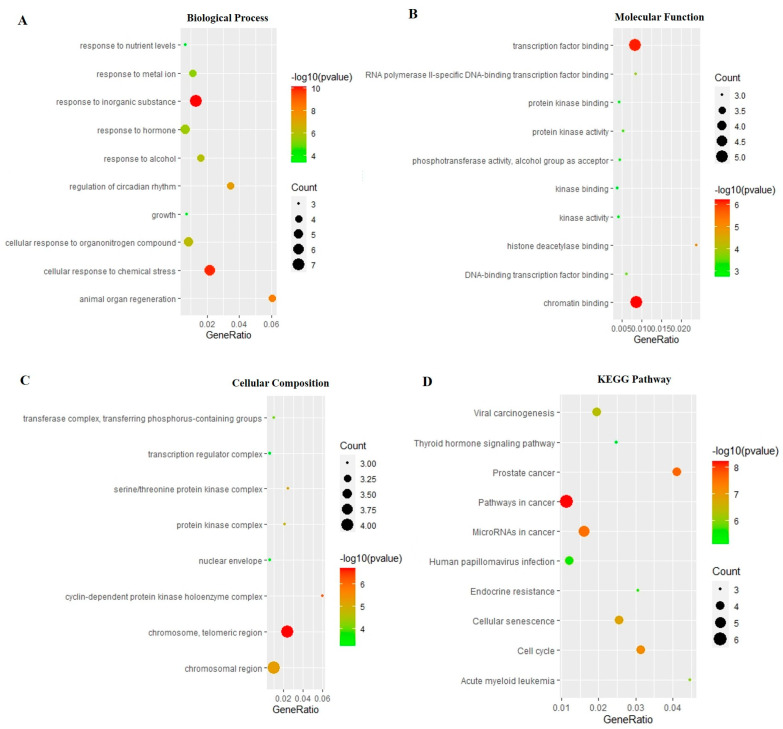
GO enrichment and KEGG pathway analysis of the top 10 key genes. (**A**) Biological processes, (**B**) molecular function, (**C**) cellular components, (**D**) pathway enrichment results at *p* < 0.01.

The top 10 key genes were used to pick out related biological processes (BP) and signaling pathways through Gene Ontology (GO) and Kyoto Encyclopedia of Genes and Genomes (KEGG) enrichment analysis (*p* < 0.01). Based on the GO enrichment analysis, BP, cellular components (CC), and molecular functions (MF) were significantly enriched (Figure 4A–C). The results show that the most significant BP includes response to an inorganic substance, cellular response to chemical stress (including cellular response to oxidative stress), animal organ regeneration, regulation of circadian rhythm, cellular response to organonitrogen compound, and so on. The MF was primarily involved in, for instance, chromatin binding, transcription factor binding, histone deacetylase binding, RNA polymerase II-specific DNA-binding transcription factor binding, and DNA-binding transcription factor binding. In addition, the CC was mainly concerned with the chromosome, telomeric region, cyclin-dependent protein kinase holoenzyme complex, chromosomal region, and serine/threonine protein kinase complex. According to the KEGG enrichment analysis, the top key genes were mainly enriched in pathways in cancer, microRNAs in cancer, cell cycle, cellular senescence, and so on (Figure 4D). The results were consistent with previous studies that MAT produced anti-aging effects by inhibiting oxidative stress and cellular senescence. Therefore, we next focused on these two aspects. There are five genes, PARP1, CDK1, EZH2, HDAC2, and MMP9, involved in the cellular response to the oxidative stress process. Through analyzing these five genes by KEGG pathway and the literature, we further focused on PARP1, which played an important role in longevity regulation and had a close relationship with cellular senescence. 

### 2.5. Molecular Docking Analysis of PARP1

**Figure 5:**
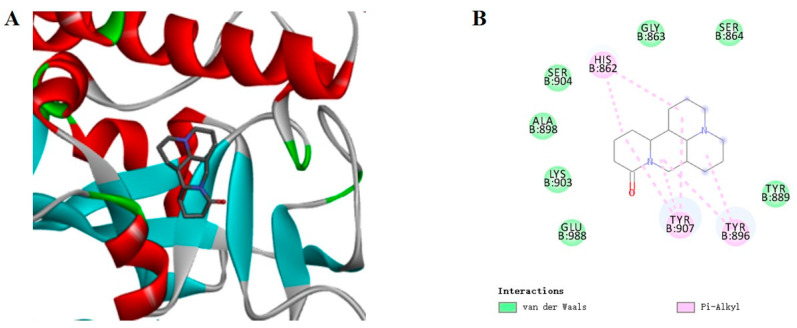
Three-dimensional and two-dimensional diagrams of the molecular docking. (**A**) Partial three-dimensional diagram of molecular docking. The protein was shown as a color ribbon, and the compound was shown as a stick. (**B**) Two-dimensional diagrams of the molecular docking.

The PARP1 was docked with the MAT. Affinity represented the binding ability calculated by the software. Generally, the affinity < −7 kcal/mol was considered a stronger binding activity [18,19]. The results showed that the affinity of PARP1 with MAT was −8.5 kcal/mol. the docking diagram of MAT and PARP1 is shown in Figure 5.

### 2.6. Molecular Dynamics Simulation of the PARP1-MAT Complex

**Figure 6:**
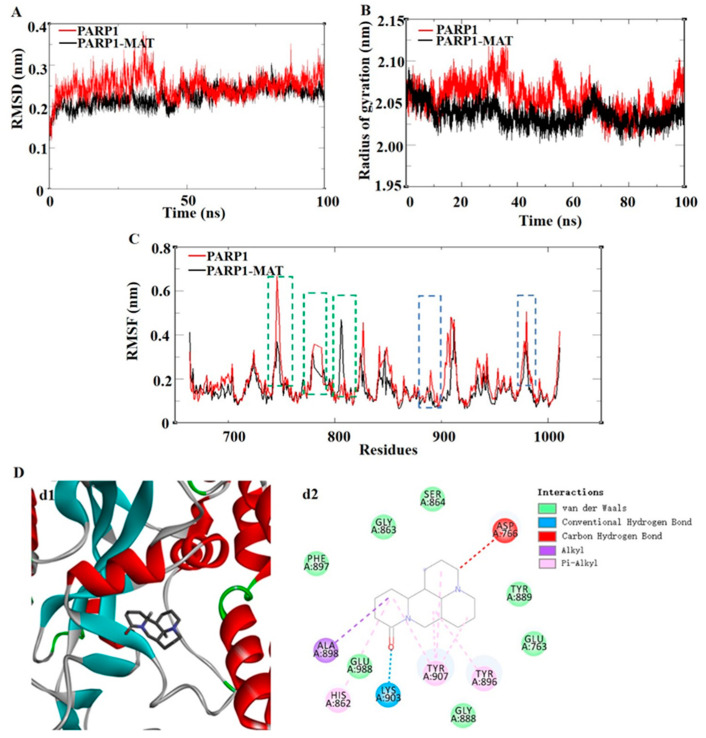
The results of molecular dynamics simulation of the PARP1-MAT complex. (**A**) RMSD of the backbone atom. (**B**) RMSF profiling of residue fluctuation, where the green boxes show the loop of protein and the blue boxes show the affinity pocket of the protein. (**C**) Rg of the backbone atoms. (**D**) Three-dimensional and two-dimensional interaction diagrams of molecular dynamics simulation of the PARP1-MAT complex, where d1 shows the 3D interaction diagram and d2 shows the 2D interaction diagram. RMSD, root mean square deviation; RMSF, root mean square fluctuation; Rg, radius of gyration.

To obtain more detailed information of the PARP1-MAT complex, the dynamic stability and structural changes in the complex systems were analyzed by the examination of root mean square deviation (RMSD), toot mean square fluctuation (RMSF), and radius of gyration (Rg). The RMSD was used to evaluate the different positions of the protein main chain at different simulation times by comparing it with the initial structure of the protein conformation. The smaller fluctuation amplitude indicates a more stable system. Figure 6A shows the RMSD curves of the skeleton atoms in the PAPR1 system (red curve) and PARP1-MAT complex system (black curve). The results show that the PARP1-MAT complex system tended to reach equilibrium around 5 ns; however, the PARP1 system tended to reach equilibrium around 40 ns. The average RMSD values in the PARP1 and PARP1-MAT systems were 0.2490 nm and 0.2241 nm, respectively. The result suggested that the PARP1-MAT complex was relatively more stable than the PARP1 alone.

The RMSF fluctuation value was calculated to reflect the protein flexibility of each residue. The average values of each RMSF were 0.1783 nm and 0.1528 nm. Compared with the PARP1 system, it was noteworthy that in these areas (residues TYR889-ASP899, 904-TYR907, and TYR986-GLU988, marked with blue-dotted boxes), the PARP1-MAT system showed obviously reduced fluctuations. Since these areas were mainly located in the affinity pocket, it demonstrated that the PARP1-MAT complex showed lower flexibility and contributed to stabilizing the protein conformation compared with PARP1 alone (Figure 6C). Rg represents the tightness of a protein structure. As shown in Figure 6B, the average Rg values in the PARP1 and PARP1-MAT systems were 2.0555 nm and 2.0360 nm, respectively. The results showed similar compactness in both structures. The complex of PARP1-MAT was primarily stabilized by van der Waals interactions, hydrogen bonds, and alkyl hydrophobic interactions and pi-alkyl hydrophobic interactions (Figure 6D).

### 2.7. The Calculation of Binding-Free Energy

The molecular mechanics/Poisson–Boltzmann surface area (MM/PBSA) algorithm was used to investigate whether the MAT affected the stability and interaction of the PARP1-MAT complex; meanwhile, the binding-free energies of the complex were calculated. The g_mmpbsa was used to calculate the binding-free energy of the simulation trajectories of the 60–65 ns intervals. As shown in Table 2, the van der Waal energy, electrostatic energy, polar solvation energy, SASA energy, and binding-free energy between PARP1 protein and MAT were −34.246 +/− 2.307, −11.513 +/− 2.152, −32.912 +/− 3.022, −3.117 +/− 0.146, and −15.963 +/− 2.784 kcal/mol, respectively. These indicated that they had a high binding affinity, and the van der Waal energy, electrostatic energy, and SASA energy were beneficial to improve the stability of the PARP1-MAT complex. After that, the free energy decomposition of the complex was performed to discover the interaction energy of a single residue. Among them, the first eight main residues in the affinity pockets of the PARP1-MAT complex with higher binding-free energies were TYR907, TYR896, GLU988, TYR889, GLU763, ASP899, ALA898, PHE897, individually (Table 3). These data were consistent with the results of RMSF that the PARP1-MAT complex showed lower flexibility and contributed to stabilizing the protein conformation compared with PARP1 alone.

### 2.8. MAT Effect on the NAD^+^ Level in the Liver of d-gal-Induced Aging Mice

**Figure 7:**
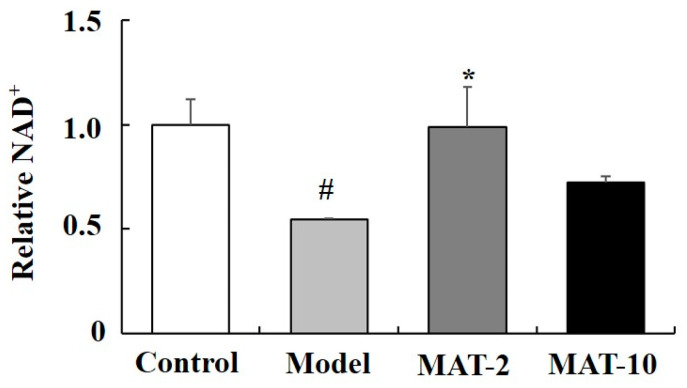
Effect of MAT on NAD^+^ level in the liver of d-gal-induced mice in vivo. Each value represents the mean ± SEM of four mice. # *p* < 0.05 indicates significant difference compared with the control group; * *p* < 0.05 indicates significant difference compared with the model group. Control group (saline + 2% ethanol in saline), model group (d-gal 150 mg/kg + 2% ethanol in saline), M-2 (d-gal 150 mg/kg + matrine 2 mg/kg), M-10 (d-gal 150 mg/kg + matrine 10 mg/kg).

Since PARP1 is one of the important consumers of nicotinamide adenine dinucleotide (NAD^+^), the MD showed that MAT may inhibit PARP1 activity. We investigated the NAD^+^ changes in the liver after being treated with MAT at different doses in the d-gal-induced aging mice. The results showed that compared with the blank control group, NAD^+^ decreased significantly in d-gal-induced aging mice. Additionally, relative to the model group, the NAD^+^ level increased in the liver of MAT at the dose of 2 mg/kg (Figure 7).

## 3. Discussion

In the present study, we collected several key targets and pathways of MAT against aging based on the network pharmacology analysis. A total of 193 targets were collected after overlapping cellular aging-related genes with potential targets of MAT. Then, the top 10 key genes were obtained after the intersection of the genes filter by three methods: MMC, MCODE, and degree. 

The top 10 key genes are PARP1, mTOR, CCND1, CDK1, CCNA2, AR, EZH2, ALB, HDAC2 and MMP9. PARP1 is overactivated and rapidly depletes the intracellular NAD^+^ and ATP pools as the extensive mtDNA is damaged [20]. The depletion of NAD^+^ and ATP pools results in cell death. The previous studies showed that PARP1 inhibition could rescue the short lifespan in hyperglycemic *Caenorhabditis elegans* (*C. elegans*) [21], and troxerutin ameliorated hepatic lipid homeostasis by decreasing PARP1 expression and increasing NAMPT expression [22]. Moreover, Guo S. et al. found that inhibition of muscle PARP1 extended the lifespan in *Drosophila* through AMPKα PARylation [23]. The inhibition of mTOR could increase the lifespan of *C. elegans*, fruit flies, yeast, and mice. mTORC1 inhibition could delay aging by stimulating autophagy, improving mitochondrial functions, clearance of senescent cells, and inhibiting senescence-associated secretory phenotype [24]. CCND1, CDK1, and CCNA2 are closely related to the cell cycle; silencing CCNA2, notably induced cellular senescence, while overexpression of CCNA2 delayed cellular senescence [25].

According to KEGG enrichment analysis, the key genes were involved in the cell cycle and cellular senescence. Cellular senescence is one of the key drivers of aging. Cellular senescence entails the arrest in the G1 phase of the cell cycle in response to damaging stimuli [26]. Decreasing the senescent cells could extend the health span of the mice and ameliorate tissue dysfunction [27]. With the exception that the senescent cells could cause various chronic diseases, such as liver cirrhosis, Alzheimer’s disease, osteoarthritis, diabetes, and so on. In addition, the senolytic could ameliorate aging-related diseases [28]. Four of the key genes involved in cellular senescence are CCND1, CCNA2, CDK1, and mTOR. The oxidative stress induced by excess reactive oxygen species (ROS), induced p21, and p16 gene overexpression, further inhibited the CCND1, CCNA2, and CDK1 expression [29], finally resulting in cellular senescence (cellular senescence, KEGG). Moreover, in our previous study, the MAT could inhibit p21 and p16 expression in the d-gal-induced aging model [13].

The key genes were mainly rich in the cellular response to the oxidative stress process in BP, and five genes, PARP1, CDK1, EZH2, HDAC2, and MMP9, were involved in the cellular response to the oxidative stress process. Oxidative stress plays an important role in aging and aging-related diseases. A suitable amount of ROS is beneficial for health; however, excess ROS can damage proteins, lipids, and DNA. As the oxidation reaction center, the mitochondrial area is one of the most important places where ROS is produced. The decrease in mitochondrial efficiency resulting in increased ROS production leads to cellular senescence and aging [30]. NAD^+^, which is famous for its role in redox reactions, participated in the regulation of biological processes widely. Fluctuations of NAD^+^ can affect redox reactions, mitochondrial function, inflammation, and so on. The studies showed that NAD^+^ levels stably declined as aging, and restoration of NAD^+^ levels could extend health span and ameliorate aging-related diseases [31]. Previous study showed that NAD^+^ supplementation improved mitochondrial function and increased the lifespan in mice [32]. PARP1 is one of the key consumers of NAD^+^. The overactivation of PARP1 could consume excessive NAD^+^ and the decreasing NAD^+^ level could inhibit the SIRT1 activity and produce the ROS, which finally results in mitochondria dysfunction and cellular senescence [33,34,35,36]. Furthermore, the suitable amount of NAD^+^ could suppress oxidative stress [37]. With aging, the PARP1 increases while DNA is damaged and the NAD^+^ level decreases significantly [21,38]. The inhibition of PARP1 overactivation could delay aging and aging-related diseases [21,39,40], and increasing the NAD^+^ level through inhibition of PARP1 or through the supplementation of NAD^+^ (or NAD^+^ precursor) also could improve lifespan [36,41]. Therefore, we focused on PARP1/NAD^+^-mediating cellular senescence signaling pathway.

In this study, the result showed that the binding affinity was −8.5 kcal/mol between PARP1 and MAT according to docking, and MD studies further proved that the binding between PARP1 and MAT was stable. Some previous studies reported that the important binding position of PARP1 inhibitors were Tyr907, Lys903, Ala898, and GLU988 [42,43]. Moreover, our results showed that the important binding positions of PARP1-MAT were TYR907, TYR896, GLU988, TYR889, GLU763, ASP899, ALA898, PHE897. This means that MAT performed a similar binding situation with the inhibitors. The previous study showed that matrine could decrease the PARP1 expression to trigger autophagy [44]. Furthermore, the in vivo results showed that MAT could increase the d-gal-induced decreased NAD^+^ levels. Therefore, the MAT could delay aging through the inhibition of PARP1 and elevation of NAD^+^ levels.

Summarily, MAT has been proven to be potentially beneficial against aging based on network pharmacology in the present study. The top 10 key genes were enriched in cellular senescence based on KEGG analysis and the cellular response to oxidative stress according to the BP. Moreover, PARP1 may be one of the most important targets for MAT in treating aging. Meanwhile, it was proven that MAT could bind to the cavity of PARP1 proteins based on molecular docking and dynamics simulation. In addition, the NAD^+^ level increased in the d-gal-induced aging model after being treated with MAT. Therefore, we proposed that the PARP1/NAD^+^-mediated cellular senescence signaling pathway may be a possible molecular mechanism for MAT in the treatment of aging.

## 4. Materials and Methods

### 4.1. Clustering of Matrine- and Aging-Related Target Genes

The chemical structure of MAT was drawn using chemical draw (CambridgeSoft, Cambridge, MA, USA) according to the previous report [45], and the pharmacological and molecular properties of MAT were obtained from the TCMSP database (https://tcmspw.com/tcmsp.php (accessed on 8 June 2022)) [46]. The previous finding showed that BBB > 0.3 was considered to be strong penetration, DL ≥ 0.18 was considered to be a drug-like compound, and OB ≥ 40% was considered to be good bioavailability [47]. TPSA less than 60 is considered to be good at permeating cell membranes. The PharmMapper [48,49] (http://www.lilab-ecust.cn/pharmmapper/ (accessed on 20 July 2022)), SwissTargetPrediction (http://www.swisstargetprediction.ch/ (accessed on 21 July, 2022)), and SuperPred [50] (https://prediction.charite.de/ (accessed on 21 July 2022)) were used to predicate the candidate target of MAT. Finally, the UniProt database (https://www.uniprot.org/ (accessed on 24 July 2022)) was used to convert all targets into gene symbols in a standard fashion. 

GeneCards (https://www.genecards.org/ (accessed on 15 October 2021)), OMIM (https://www.omim.org/ (accessed on 15 October 2021)), and the NCBI GENE database (https://www.ncbi.nlm.nih.gov/gene/ (accessed on 15 October 2021)) were utilized to screen the senescence-related genes. The keywords “cellular senescence” and “aging” were used to screen the targets. Next, the targets from GeneCards were selected if the GIFtS value was greater than 40 [51]. Subsequently, the targets of MAT were mapped to aging-related targets and then potential targets of MAT against aging were obtained through the Venn 2.1 (https://bioinfogp.cnb.csic.es/tools/venny/ (accessed on 30 July 2022)) intersection.

### 4.2. The Protein–Protein Interaction Network Map Construction of Potential Target Genes of Matrine against Aging

The STRING database (https://string-db.org/ (accessed on 15 August 2022)) was used to create the PPI Network. The homo species and the minimum confidence level at 0.4 were chosen for analysis. Next, the result was edited by using Cytoscape software (version 3.7.1). The MMC, MCODE algorithms [52], and DC were used to screen key genes. The MCODE was utilized to explore the functional gene clusters, and the genes in the first functional module with the highest MCODE score were selected. The top 20 node genes were obtained by calculating the MCC. The genes whose DC value was greater than twice the median of DC were selected. Finally, the intersection of these genes obtained from the three methods (MCODE, MCC, and DC) was taken as the key genes, and the result was displayed via a Venn diagram [53]. 

### 4.3. GO and KEGG Pathway Enrichment Analysis

To explain signal pathways and the function of MAT, Metascape (http://metascape.org/gp/index.html (accessed on 5 October 2022)), which is the functional annotation clustering tool, was used to analyze the KEGG pathway enrichment and the GO function of the top 10 key genes of MAT acting on aging. Meanwhile, the signal pathways, the BP, CC, and MF associated with the key genes of MAT against aging were described. 

### 4.4. Molecular Docking

To verify the affinity between MAT and the PARP1, the molecule docking was performed by using Auto Dock software (version 1.5.6). The protein (4zzz) was downloaded from the PDB (https://www.rcsb.org/ (accessed on 5 October 2022)). Next, the protein was modified as followed: removing water molecules and ligands, adding hydrogen and charge; setting up the docking grid box at the center of the molecule. The MAT was modified as follows: minimizing the energy by ChemDraw 3.0 (CambridgeSoft, Cambridge, MA, USA), adding the charge, and then setting the rotatable key. Finally, the binding conformation with the lowest energy was chosen and the binding diagram was observed by using Discovery Studio (BIOVIA, Shenzhen, China).

### 4.5. Molecular Dynamics Simulations

After molecular docking, the PARP1–MAT complex or PARP1 protein alone was subjected to MD simulations using the GROMACS 2019.6 package with GROMOS96 54A7 force field, and the SPC water model [54], the MAT was subjected to Automated Topology Builder (ATB) and Repository (Version 3.0) to obtain the ligand topology files. The charge in the system was neutralized by adding a chloride ion. Energy minimization was carried out with the steepest descent minimization using the Verlet cut-off-scheme. After the equilibration in the NVT ensemble and in the NPT ensemble, the simulations were performed for 100 ns at a constant pressure of 1 bar and a constant temperature of 300 K [55]. The compressed coordinates in the PARP1-MAT complex or PARP1 system were saved at 10-ps intervals throughout the simulations. The statistical prediction for RMSD, RMSF, and Rg was analyzed using GROMACS [56].

### 4.6. Binding-Free Energy Calculation

We used the Open Source Drug Discovery Software g_mmpbsa (version 1.1.0) to calculate the binding-free energy base on MM/PBSA calculations [57]. Five hundred snapshots of the PARP1-MAT complex were taken from 60 to 65 ns time intervals to compute the MM/PBSA. The potential energy (Van der Waals interactions + electrostatic), the free solvation energy (polar + non-polar solvation energies), and the total binding-free energy of the PARP1-MAT complex were calculated, and the free energy decomposition of the complex was performed.

### 4.7. Animals and Drug Administration

The 8-week-old male Institute of Cancer Research mice (weighing 28–30 g) were purchased from Shanxi Medical University (Shanxi, China). The mice were fed and provided access to food and water freely and housed in constant temperature (23 ± 1 °C) and relative humidity (55 ± 5%) under 12 h:12 h light/dark cycle. The mice were fed to the adaption to the environment for a week before the experiment. The mice were randomly divided into four groups as follows (six mice in one group): the blank control group, model group (150 mg/kg d-gal), MAT low-dose group (2 mg/kg MAT), and PAE high-dose group (10 mg/kg MAT). For the first six weeks, the blank control group was injected once daily with saline, and the other groups were treated with once-daily d-gal (150 mg/kg) by subcutaneous injection. From the fifth week, the MAT groups were subjected to MAT (at doses of 2 and 10 mg/kg, oral) once daily for five weeks, and the blank control group and model group were administered to solvent (2% ethanol in oil, oral) in the same way instead. All experimental procedures were conducted and performed following the policies for animal care and use encompass regulations approved by the Institutional Animal Care and Use Committee of Shanxi Agricultural University (SXAU-EAW-2019M002004, 12 March 2019).

### 4.8. NAD^+^ Analysis

Before detection, the liver tissues were rapidly homogenized in an acid-extracting solution (10 times the tissue weight) with a homogenizer. The homogenates were boiled for 5 min and then centrifuged at 10,000× *g* at 4 °C for 10 min; the supernatant was collected and then an equal volume of alkaline-extracting solution was added to it. The mixture was centrifuged at 10,000× *g* at 4 °C for 10 min, and the supernatant was collected for detection. The NAD^+^ content was determined with an NADH/NAD^+^ assay kit (Solarbio Science & Technology Co., Ltd., Beijing, China) following the manufacturer’s protocols. The protein concentration was determined by the Bradford protein assay kit (Solarbio Science & Technology Co., Ltd., Beijing, China). The NAD^+^ content was measured at 570 nm. 

### 4.9. Statistical Analyses

The results of the relative NAD^+^ were expressed as mean ± SEM. Data were analyzed by one-way analysis of variance analysis. Statistical significance was set at *p* < 0.05.

## Figures and Tables

**Table 1:** Pharmacological and molecular properties of matrine.

Name	MW	AlogP	TPSA	HL	DL	OB (%)	BBB
MAT	248.41	1.42	23.55	6.69	0.25	63.77	1.52

Legend: MW, molecular weight; AlogP, the Ghose-Crippen octanol-water partition coefficient; BBB, blood-brain barrier; DL, drug-likeness; HL, drug half-life; OB, oral bioavailability; TPSA, topological polar surface area.

**Table 2:** The entropic distribution of the total binding-free energy for the PARP1-MAT Complex.

van der Waal Energy (kcal/mol)	Electrostattic Energy (kcal/mol)	Polar Solvation Energy (kcal/mol)	SASA Energy (kcal/mol)	Binding Energy (kcal/mol)
−34.246 +/− 2.307	−11.513 +/− 2.152	−32.912 +/− 3.022	−3.117 +/− 0.146	−15.963 +/− 2.784

**Table 3:** The top eight main residues in PARP1 that contribute to the binding-free energy of PARP1 and MAT.

Name	TYR907	TYR896	GLU988	TYR889	GLU763	ASP899	ALA898	PHE897
Energy (kJ/mol)	−10.965	−7.3233	−5.4298	−5.3366	−2.9389	−1.8721	−1.2314	−0.991

## Data Availability

Not applicable.
